# Supplementary material for: Engineering of a Long-Acting Bone Morphogenetic Protein-7 by Fusion with Albumin for the Treatment of Renal Injury
Source: Pharmaceutics. 2022 Jun 24;14(7):1334. doi: 10.3390/pharmaceutics14071334 (PMC9316787; doi:10.3390/pharmaceutics14071334)
Supplement: Supplementary file 1 [file pharmaceutics-14-01334-s001.zip › pharmaceutics-1763949-supplementary.pdf]

## Supplemental Methods

### 1. Materials and Methods

- **Production of an HSA–BMP7 fusion protein**

The genetic fusion of BMP7 and HSA was performed, and the fusion protein was produced following a previously reported method [17,18]. Unglycosylated BMP7 (N10Q, N29Q, and N80Q) cDNA, which contains restriction sites AvaI and EcoRI at 5′-terminal and 3′-terminal, respectively, was purchased from the Biomatik Company (Ontario, Canada). The BMP7 cDNA fragment was digested with AvaI and EcoRI and ligated into the 3′-terminal end of HSA in a pPIC9–HSA plasmid [17,18] digested with AvaI and EcoRI to give pPIC9–HSA–BMP7. The N-terminal region of BMP7 is linked via a linker polypeptide (Gly-Gly-Gly-Gly-Ser-Gly-Gly-Gly-Gly-Ser) to the C-terminal region of HSA. After digestion of the pPIC9–HSA–BMP7 with the restriction enzyme SalI, the transformation was achieved by homologous transformation to the HIS4 gene locus of Pichia pastoris (GS115 strain, Invitrogen, Carlsbad, CA, USA) using an electroporation device (Gene Pulser 11 Electroporation System, BIO-RAD).

Regarding the expression and purification of the fusion protein, the transformed Pichia pastoris cells were incubated in 5 L of BMGY liquid media (1% yeast extract, 2% peptone, 100 mM potassium phosphate (pH 6.0), 1.34% yeast nitrogen base with ammonium sulfate without amino acids, 4 × 10-5 % biotin, and 3% glycerol) (growth phase) for 2 days (OD600 = 2) and then cultured in 800 mL of BMMY media that contained a protein expression inducer as well as a carbon source, methanol (1% yeast extract, 2% peptone, 100 mM potassium phosphate (pH 6.0), 1.34% yeast nitrogen base with ammonium sulfate without amino acids, 4 × 10-5 % biotin, and 1.5% methanol) (protein induction phase) for 3 days at 30 °C. Methanol was added daily, to permit the concentration of methanol to be maintained at a level of 1.5% in order to sustain the induction effect for protein expression. Purification of the fusion protein was initially carried out via chromatography on a Blue Sepharose 6 Fast Flow column (GE Healthcare, Tokyo, Japan) equilibrated with 200 mM sodium acetate buffer (pH 5.5) after dialysis against the same buffer. Using AKTA prime, a 5 mL HiTrap Phenyl HP column (GE Healthcare, Tokyo, Japan) was then used for hydrophobic chromatography, under the following conditions: Buffer A, 50 mM Tris–HCl + 1.5 M ammonium sulfate, pH 7.0; Buffer B, 50 mM Tris–HCl, pH 7.0; gradient, 0-100% (Buffer B) 100 mL; flow rate, 5 mL/min.

The desired fusion protein was analyzed with SDS–PAGE using a 15% polyacrylamide gel with Coomassie blue R250 staining. The fusion protein was estimated to be more than 95% pure. Western blotting was performed using a mouse anti-BMP7 antibody (Santa Cruz Biotechnology, Inc., Santa Cruz, CA, USA), followed by a secondary antibody of goat anti-mouse IgG conjugated to horseradish peroxidase (HRP), or using a rabbit anti-HSA antibody (Sigma-Aldrich, Inc., St. Louis, MO, USA), followed by a secondary antibody of goat anti-rabbit IgG conjugated to HRP.

- **Circular dichroism spectral measurement**

CD spectra were measured using a JASCO J-820 spectropolarimeter (JASCO, Tokyo Ja-pan) at 25 °C. Far-UV CD spectra were recorded at protein concentrations of 2.5 μM in 67 mM phosphate buffer. The CD intensity was expressed as molar ellipticity.

- **In vitro activity of HSA–BMP7**

To evaluate the effect of HSA–BMP7 on the phosphorylation of Smad1/5/8 in human kidney (HK-2) cells, HK-2 cells were incubated in 6-well plates in a K-SFM medium containing 5 ng/mL of human recombinant EGF and 0.05 µg/mL of bovine pituitary extract at 37 °C for 24 hr and then incubated with glycosylated BMP7 that was derived from CHO cells (10 nM) or HSA–BMP7 (250 nM or 500 nM) for 60 min in K-SFM. The phosphorylation of Smad1/5/8 protein HK-2 cells was determined via Western blotting using a rabbit anti-p-Smad 1/5/8 antibody (Santa Cruz Biotechnology, Inc., Santa Cruz, CA, USA).

To evaluate the effect of HSA-BMP7 on the TGF-β-stimulated mRNA expression of α-smooth muscle actin (α-SMA) in HK-2 cells, the level of α-SMA mRNA in HK-2 cells was determined using quantitative RT-PCR in the presence of 3 ng/mL TGF-β with/without glycosylated BMP7 derived from CHO cells (10 nM) or HSA-BMP7 (500 nM) for 48 hr. Total RNA was extracted from the cells using RNAiso PLUS (TaKaRa Bio Inc., Shiga, Japan) according to the manufacturer's protocol. The concentration and the purity of the RNA extract were determined by measuring the absorbance at 230 nm, 260 nm, and 280 nm. The cDNA was synthesized using PrimeScript® RT master mix (Perfect Real Time) (TaKaRa Bio Inc.). Quantitative real-time RT-PCR analyses were performed in an iCycler thermal cycler (Bio-Rad) with an iQ5 qRT-PCR detection system attached (Bio-Rad) using SYBR® Premix Ex TaqII (Perfect Real Time) (TaKaRa Bio Inc.). PCR amplifications were performed under the following conditions: 95 °C for 3 min, for 40 cycles at 95 °C for 10 s (denaturation step), and at 60 °C for 1 min (annealing/extension steps). The following primer sequences were used: human α-SMA (forward: 5′-CGGGACATCAAGGAGAAACT-3′, reverse: 5′-GCCCATCAGGCAACTCGTAA -3′), and human GAPDH (forward: 5′-GGTGAAGGTCGGAGTCAACG-3′, reverse: 5′-ACCATGTAGTT-GAGGTCAATGAAGG-3′).

To evaluate the effect of HSA–BMP7 on osteogenic activity, the alkaline phosphatase (ALP) activity in mouse myoblast cells (C2C12 cells) was determined. C2C12 cells were incubated in 24-well plates in DMEM with 10% heat-inactivated FBS at 37 °C for 24 hr. Glycosylated BMP7 derived from CHO cells, unglycosylated BMP7 derived from E. coli., or HSA–BMP7 was added to the DMEM with 5% FBS. After incubating the cells for 9 days, ALP activity was determined by using LabAssayTM ALP (Wako Pure Chemical, Osaka, Japan).

- **Pharmacokinetic analyses of HSA–BMP7**

A 125-Iodinated fusion protein was prepared based on our previously reported procedures and purified using a PD-10 column (GE Healthcare, Inc. CA, USA) [17]. The recovered $^{125}$I-fusion had a specific activity of $2.0 \times 10^7$ cpm/µg and was diluted with non-labeled fusion to adjust the dose (mg/kg) of fusion protein before being administered to mice.

All animal experiments were undertaken in accordance with the guideline principle and procedures of Kumamoto University for the care and use of laboratory animals (No. A 2021- 021). All animals were maintained in a temperature-controlled room with a 12 hr dark/light cycle with ad libitum access to food and water. Male ICR mice (3 weeks, male, Japan SLC Inc., Shizuoka, Japan) were allowed to acclimate for one week (4 weeks, 28–30 g) before being used in the experiments. The $^{125}$I-protein ($15 \times 10^4$ cpm/30 g mouse; 0.1 mg/kg) was injected into the tail vein of male ICR mice. A mouse was killed at various time points (1, 5, 10, 30, 60, 120, 360, and 720 min), and approximately 500 µL samples of blood and plasma were obtained via centrifugation at 3000×g for 10 min. Aqueous tri-chloroacetic acid (TCA, 25%, 0.1 mL) was first added to the plasma (20 µL) diluted with 5 g/dL HSA (80 µL), followed by centrifugation (3000×g, 10 min). The precipitate was then washed with 12.5% TCA (0.2 mL), and the radioactivity of the pellet was measured. The

radioactivity contained in the samples was determined using a $\gamma$-counter (ARC-5000; Aloka). Pharmacokinetic parameters were calculated based on moment analysis (non-compartment model).

- **Mouse model of unilateral ureteral obstruction (UUO)-induced renal fibrosis**

The anti-fibrotic effect of HSA–BMP7 was evaluated in mice with unilateral ureteral obstruction (UUO). The ICR mice underwent unilateral ureteral ligation at 4 weeks of age. Two ligatures, 5 mm apart, were placed in the upper two-thirds of the ureter. Saline, HSA–BMP7, HSA, or glycosylated BMP7 derived from CHO cells (100 nmol/kg) was then administered intravenously just after and at 7 days after the UUO. At 14 days after the UUO, the mice were sacrificed under anesthesia. The obstructed kidney was removed, and an upper part of the kidney was homogenized for the Western blotting analysis, the middle part of the kidney was used for quantitative RT-PCR, and the lower part of the kidney was fixed in phosphate-buffered 10% formalin and prepared for routine histological examination.

- **Histopathological analysis**

The removed kidney that was fixed in 10% phosphate-buffered formalin was dehydrated using a graded series of ethyl alcohol concentrations and then embedded in paraffin. Kidney blocks were cut into 2 μm sections, which were dewaxed in xylene, rehydrated in decreasing concentrations of ethanol, and washed in water for 5 min.

For Masson's trichrome staining, the sections were sequentially treated with solution A (5% (wt/vol) potassium dichromate and 5% (wt/vol) TCA), Weigert's iron hematoxylin, solution B (1.25% (wt/vol) phosphotungstic acid and 1.25% (wt/vol) phosphomolybdic acid), 0.75% (wt/vol) Orange G solution, solution C (0.12% (wt/vol) xylidine ponceau, 0.04% (wt/vol) acid fuchsin, and 0.02% (wt/vol) azophloxin), 2.5% (wt/vol) phosphotungstic acid, and finally an aniline blue solution. Masson's trichrome staining samples were mounted with malinol and inspected with the aid of a microscope (Keyence, BZ-8000, Osaka, Japan).

For the immunostaining of α-SMA, antigen retrieval was first conducted using HistoVT One. A solution containing 50 mM Tris–HCl + 0.1% Tween-20 (T-TB) was then used to solubilize the kidney sections, followed by blocking with Block Ace at room temperature for 20 min. The primary antibody reaction was then conducted overnight at a temperature below 4 °C. The kidney sections were then washed with T-TB, followed by a reaction with the secondary antibody at room temperature for 1.5 h. The stained sections were then observed using a microscope. The following antibodies were used in this study: mouse anti-α-SMA antibody (Santa Cruz Biotechnology, Inc., CA, USA) and Alexa Fluor® 488 goat anti-mouse IgG (H + L) secondary antibody (Life Technologies, Inc., CA, USA).

- **Determination of hydroxyproline levels**

The hydroxyproline content was determined as described previously [19]. On day 14 after the UUO, the kidney was removed and homogenized in 1 ml of 50% TCA. After centrifugation, the pellets were hydrolyzed in 0.5 ml of 10 N HCl for 16 hr at 110ºC. Each sample was incubated for 20 min at room temperature after the addition of 0.5 ml of a 1.4% (wt/vol) chloramine T solution and then incubated at 65ºC for 15 min after the addition of 0.5 ml of Ehrlich's reagent (1 M DMBA, 70% (vol/vol) isopropanol and 30% (vol/vol) perchloric acid). The absorbance was measured at 540 nm to determine the amount of hydroxyproline.

- **Quantitative RT-PCR analysis**

The total RNA of the kidney was extracted using RNAiso PLUS. The primers used are shown below. Mouse collagen 1a2 (Col1a2) primers (forward: 5′-CACCCCAGCGAAGAACTCATA-3′, reverse: 5′-GCCACCATT-GATAGTCTCTCCTAAC-3′); α-SMA primers (forward: 5′-AGCCATCTTTCATTGGGATGG-3′, reverse 5′-CCCCTGACAGGACGTTGTTA-3′); TGF-β1 primers (forward: 5′-GGATACCAACTATTGCTTCAGCTCC-3′, reverse: 5′-AGGCTCCAAATATAGGGGCAGGGTC); and GAPDH primers (forward: 5′-AACTTTGG-CATTGTGGAAGG-3′, reverse: 5′-ACACATTGGGGGTAGGAACA-3′).

- **Cisplatin-induced acute kidney injury model**

Male ICR mice at 4 weeks of age were randomized to receive saline or HSA–BMP7 (200 nmol/kg) 30 min before the intraperitoneal administration of cisplatin (15 mg/kg). Controls were injected with only saline. HSA–BMP7 was injected via the tail vein. The mice were allowed to recover in metabolic cages from 72 hr to 96 hr after the administration of cisplatin, and urine samples were collected and used for the determination of urinary creatinine excretion. After collecting blood samples for the determination of serum creatinine (SCr) and blood urea nitrogen (BUN), the mice were sacrificed 96 hr after the administration of cisplatin. The kidneys were fixed in phosphate-buffered 10% formalin and prepared for routine histological examination.

- **Biochemical evaluation of blood and urine samples**

SCr and urinary creatinine concentrations were measured via enzymatic methods, using the respective assay kits (Wako Pure Chemical, Osaka, Japan). BUN was determined via the diacetylmonoxime method using an assay kit (Wako Pure Chemical). Creatinine clearance during 24 hr (from 72 hr to 96 hr) after the injection of cisplatin was calculated as mL/min.

- **Histologic examination of renal tissues**

The left kidney fixed in 10% formalin was dehydrated in a graded series of ethanol concentrations and embedded in paraffin. Kidney blocks cut into 2 μm sections were subjected to periodic acid–Schiff (PAS) staining for morphologic analysis and TUNEL staining for cell apoptosis. PAS-stained tissue sections were viewed using light microscopy at a x200 magnification. For the semi-quantitative analysis of morphological changes, 20 high-magnification (x400) fields of the cortex and the outer stripe of the outer medulla in mice were randomly selected. The injury ratio (extent of degeneration, detachment, tubulitis, and dilatation of tubular cells/total tubular cells x100) was then measured. All quantifications were performed in a blind manner. For TUNEL staining, sections were stained using an in situ cell death detection kit from Fluorescein (Roche, Basel, Switzerland), according to the manufacturer's protocol, for paraffin-embedded sections. The slide was observed using a microscope.
